# Characteristics of prefrontal activity during emotional and cognitive processing in patients with bipolar disorder: A multi-channel functional near-infrared spectroscopy study

**DOI:** 10.3389/fnins.2022.946543

**Published:** 2022-07-26

**Authors:** Mengchai Mao, Guifang Chen, Kun Feng, Dongsheng Xu, Xuyun Hua, Chunlei Shan, Pozi Liu

**Affiliations:** ^1^Engineering Research Center of Traditional Chinese Medicine Intelligent Rehabilitation, Ministry of Education, School of Rehabilitation Science, Shanghai University of Traditional Chinese Medicine, Shanghai, China; ^2^YuQuan Hospital, School of Clinical Medicine, Tsinghua University, Beijing, China; ^3^Affiliated Hospital of Zunyi Medical University, Guizhou, China; ^4^Center of Rehabilitation Medicine, Yueyang Hospital of Integrated Traditional Chinese and Western Medicine, Shanghai University of Traditional Chinese Medicine, Shanghai, China

**Keywords:** bipolar disorder, emotion, verbal fluency, functional near-infrared spectroscopy, prefrontal cortex

## Abstract

Bipolar disorder (BD) is a recurrent chronic mental disorder with a broad profile of functional deficits including disturbed emotional processing and cognitive impairments. The goal of the current study was to further explore the underlying neural mechanism of dysfunction in patients with BD from a comprehensive perspective of both cognition and emotion. Forty-six clinical patients with BD and forty-five healthy controls performed emotion induction task and verbal fluency task (VFT), with frontal activity measured by functional near-infrared spectroscopy (fNIRS). Our results show distinct hemodynamic activity in the prefrontal region during emotional and cognitive processing between patients with BD and healthy controls. Patients with BD exhibit valence-dependent prefrontal cortex (PFC) hemodynamic response to emotional stimuli, with bilateral frontal hypoactivity indicating decreased positive reactivity and left frontal hyperactivity indicating increased negative reactivity. On the other hand, patients with BD showed impaired performance with bilateral frontal hypoactivity during VFT. Taken together, frontal dysfunction of cognition and emotionality in patients with BD probed by fNIRS would be a potential biomarker in clinical assessment.

## Introduction

Bipolar disorder (BD) is a recurrent chronic mental disorder characterized by mood and energy fluctuations between manic and depressive episodes (Grande et al., 2016). Among mood disorders, BD has the highest risk of suicide (Gonda et al., 2012). It affects over 1% of the world population, with an estimated lifetime prevalence of 2.5% (Merikangas et al., 2011), and leads to high rates of morbidity and mortality, especially among young and working-aged people (Alonso et al., 2011).

Patients with BD exhibit a broad profile of functional deficits (Vieta et al., 2018), including disturbed emotional processing and cognitive impairments (Bourne et al., 2013; Douglas et al., 2018). Emotion dysfunction in patients with BD manifests in heightened or more frequent negative affectivity measured by self-report (Heerlein et al., 1998) and standard assessment (Pavlova et al., 2011). Among cognitive impairments, key deficits lie in executive function, attention, verbal memory, and non-verbal memory (Lima et al., 2018). An overall meta-analysis reveals functional impairment in verbal fluency in participants with BD compared to healthy controls (Raucher-Chene et al., 2017). In addition, a longitudinal study shows that verbal fluency deficits were more prominent with depressive symptoms (Chaves et al., 2011).

In recent years, there is an increasing number of studies investigating potential biomarkers of BD using neuroimaging techniques (Teixeira et al., 2019), largely magnetic resonance imaging (MRI). Structural alternation and abnormal functional activation of BD when compared to a healthy group were reported in a series of MRI studies (Chen et al., 2011; De Peri et al., 2012; Ambrosi et al., 2017; Waller et al., 2021). Despite the fruitful results achieved from MRI studies, MRI experiments are confined to the scanning room, with little susceptibility to movements. These disadvantages led to a limited clinical application of MRI in BD research and validated biomarkers for BD in clinical practice, which could aid diagnostic accuracy and allow for early intervention and prognosis across the lifespan, and remains to be further investigated (Grande et al., 2016).

Functional near-infrared spectroscopy (fNIRS) is an emerging non-invasive neuroimaging technique that has attracted increasing attention in the past 30 years (Boas et al., 2014). It sheds near-infrared light into the outer layer of the cerebral cortex and the light absorption varies with the change in hemoglobin concentration induced by cortical activities (Bunce et al., 2006). fNIRS has an acceptable spatial and temporal resolution, good portability, and little restriction of body movements, allowing for various kinds of experiments including near-natural circumstances (Boas et al., 2014). Given these unique properties, fNIRS has become a well-established tool for neuroscience research, used not only for neural activity in healthy populations but also as a probe for assessment in clinical application (Fujimoto et al., 2014; Tsujii et al., 2018; Ma et al., 2020; Devezas, 2021).

Currently, fNIRS-based BD studies focus largely on cognitive processing. Reduced frontal activation was observed during various cognitive processing with compromised performance (Takei et al., 2014; Ono et al., 2015; Fu et al., 2018; Tsujii et al., 2018; Zhu et al., 2018; Chen et al., 2021), regardless of the specific mental status of the patients. Yet little attention was paid to emotion processing in fNIRS-based BD research, and results were inconsistent. Matsubara et al. (2014) reported reduced activity in the superior and middle frontal regions to happy words, and hyperactivity in the left inferior frontal region to threat words. Aleksandrowicz et al. (2020) found a decreased activity of emotional words, regardless of the valance, in the frontal and frontotemporal cortex in individuals at high risk for BD. Contrary, Segar et al. (2021) found an increased activity to emotional stimuli of bilateral dorsolateral prefrontal cortex (DLPFC) in risky individuals. In contrast, Kameyama et al. (2006) failed to identify a correlation between depression severity and frontal lobe dysfunction detected by fNIRS in patients with BD. To sum up, there are still unclear aspects regarding frontal lobe dysfunction in patients with BD.

To further explore the underlying neural mechanism of dysfunction in patients with BD from a comprehensive perspective of both emotion and cognition, we employed fNIRS to investigate the hemodynamic patterns of patients with BD and healthy controls during verbal fluency task (VFT) and emotion induction. We expected frontal hypoactivity during cognitive processing and a distinct pattern of frontal activity during emotional processing.

## Materials and methods

### Participants

Forty-six clinical patients with BD exhibiting depressive episodes were recruited from Yuquan Hospital. The inclusion criteria were as follows: meeting the diagnostic and statistical manual of mental disorders, fourth edition, text revision (DSM-IV-TR) diagnosis of BD, aged between 18 and 60, having more than 9 years of education, not having a history of neurological disease or chronic substance abuse (addictive drugs such as methamphetamine, ecstasy, k-powder, heroin, and alcohol). Forty-five healthy controls (HC), matched in age, sex, and education level were recruited from the local community. All participants were right-handed. The study was carried out with the written consent of each participant and was in accordance with the ethical standards of the Declaration of Helsinki. This study was approved by the Ethics Committee of Yuquan Hospital and registered at http://www.medresman.org.cn/, with Reg No. ChiCTR2100043338.

### Clinical assessments

The Hamilton Depression Scale (HAMD; Hamilton, 1960) and the Hamilton Anxiety Rating Scale (HAMA; Hamilton, 1959) were rated by an independent physician to assess the depression and anxiety states of the participants.

### Procedure

The experiment was carried out in a quiet room with soft lighting, and participants were seated comfortably in front of a monitor. The participants were asked to perform two tasks, emotion induction task and VFT, during fNIRS scanning, and to avoid unnecessary movement, head movement in particular, to reduce fNIRS data artifacts. The stimuli were presented Eprime 2.0.

#### Emotion induction task

**FIGURE 1:**
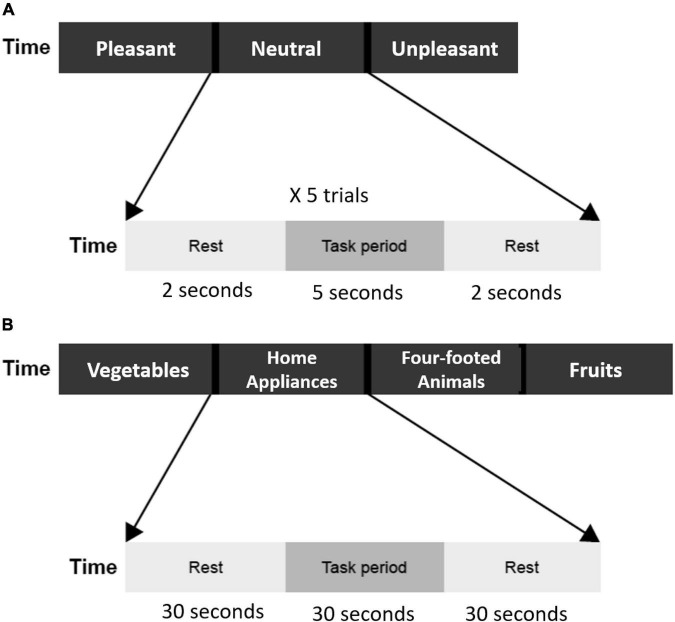
**(A)** Flow of emotion induction task. Three emotion blocks (pleasant, neutral unpleasant). Each block includes five trials, and each trial contains a 5-s task period and a 2-s rest period. The total duration of the emotion induction task was 105 s. **(B)** The flow of verbal fluency task. Four category blocks (vegetables, home appliances, four-footed animals, and fruits). Each block contains a 30-s task period and a 30-s rest period. The total duration of the verbal fluency task was 240 s.

The emotion induction task began with instruction and a fixation mark to remind participants of the upcoming task. Afterward, 15 affective pictures selected from the International Affective Picture System (Lang, 1997), 5 pleasant (mean valence 2.40 ± 0.31, mean arousal 1.43 ± 0.28), 5 neutral (mean valence 4.83 ± 0.23, mean arousal 1.02 ± 0.27), and 5 unpleasant (mean valence 7.75 ± 0.25, mean arousal 1.41 ± 0.25) were presented. Pleasant pictures consisted of esthetically pleasing, endearing, and positive (e.g., sports, cute animals) content. Neutral pictures included mundane scenes and daily objects. Unpleasant pictures consisted of disgust, sad, and threat scenes. Each picture was presented for 5 s, and participants were asked to view the pictures carefully and immerse themselves in the scenes as the picture was presented, and press keys to assess their feelings toward the picture as pleasant, neutral, or unpleasant. Then, there was a 2-s rest period with a fixation mark presented on the screen (Figure 1A).

#### Verbal fluency task

A semantic category version of the VFT was used. There were four blocks in total, each block for one category: vegetables, family applications, four-footed animals, and fruits. Each block included 30 s of task period and 30 s of a rest period. During the task period, participants were asked to speak out aloud as many examples as possible according to the category word presented on the screen. Audio responses were recorded and then transcribed for further analysis (duplicates and errors were excluded). During the rest period, a fixation mark was presented on the screen, and the participants were asked to keep quiet and relaxed (Figure 1B).

### Functional near-infrared spectroscopy measurements

**FIGURE 2:**
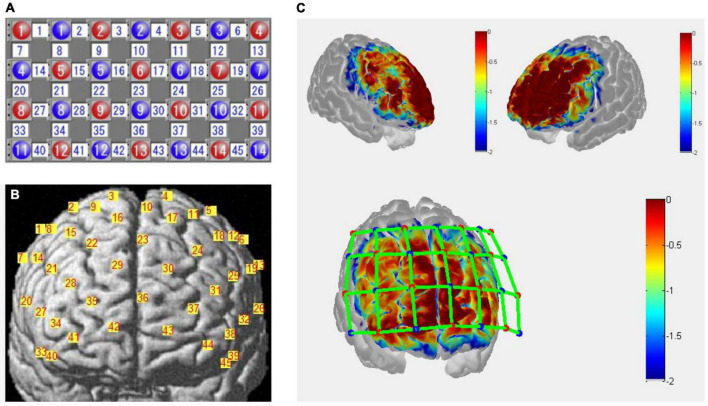
**(A)** Optodes arrangement of functional near-infrared spectroscopy (fNIRS) measurements. Numbers in red represent emitters, blue represent detectors, and white represents channels. **(B)** Channels projected to the rendered brain by a probabilistic registration process using NIRS-SPM (Ye et al., 2009; Tsuzuki and Dan, 2014). **(C)** Sensitivity map with front and side views over the frontal cortex estimated by Monte-Carlo simulation using the Atlas Viewer from Homer2 (Boas et al., 2002; Aasted et al., 2015). Red dots represent the emitters, blue dots the detectors, and green lines the channels. The colormap represents spatial sensitivity values ranging from –2 to 0 in log10 units.

The fNIRS measurements were conducted with a 45-channel continuous-wave fNIRS system (FOIRE3000, Shimadzu Co., Japan) with 14 emitters and 14 detectors (inter-optode distance, 30 mm) placed upon the frontal cortex, based on the international 10–20 system. Probes of the lowest were positioned along the Fp1-Fp2 line with the middle optode placed between channel 42 and 43 at position FPz. Probes and channel layouts are illustrated in Figure 2. Scalp positions of each optode and each channel were recorded using a 3-dimensional magnetic digitizer (PATRIOT, Polhemus Inc.) on one of the participants, to estimate the cortical locations of the corresponding channels by a probabilistic registration process using NIRS-SPM v.3.2 (Ye et al., 2009; Tsuzuki and Dan, 2014). Relative changes in concentrations of oxygenated hemoglobin (HbO), deoxygenated hemoglobin (HbR), and total hemoglobin (HbT) were calculated from the absorption of near-infrared light at three wavelengths (780, 805, and 830 nm), using the modified Beer–Lambert law.

### Functional near-infrared spectroscopy data analysis

The fNIRS data were preprocessed and analyzed using NIRS-KIT (Hou et al., 2021) based on MATLAB 2013b (The MathWorks Inc., MA, United States). Considering that the HbO signal is widely used in psychiatric studies (Ozawa, 2021), with better sensitivity to task-related hemodynamic changes (Bendall et al., 2016; Yeung and Lin, 2021), and found fair to excellent reliability at map-wise and cluster-wise scales in emotion processing involving prefrontal cortex (PFC) in a preliminary test-retest study (Huang et al., 2017), we focused on the HbO signal in the present study.

A wavelet transformation was performed to minimize the impact of motion artifacts on the functional data (Molavi and Dumont, 2012). The wavelet coefficient exceeding 1.5 times the interquartile range, which was properly due to motion artifacts, was set to zero. A first-order detrend was applied to remove linear trends from fNIRS data. Data were then bandpass filtered between 0.01 and 0.2 Hz with a third-order IIR filter to attenuate slow drifts, physiological interference, and high-frequency noises.

The General Linear Model was used to detect the hemodynamic activities of the HbO signals from each participant. The design matrix consisting of four boxcar regressors (three for emotion categories of pleasant, neutral, and unpleasant, and one for VFT) was convolved with a Gaussian HRF to obtain the predictors of the time series of brain activation. Beta-estimates of each regressor represented the weight of each condition to the variance of the hemodynamic signal. Condition-wise effects of interest were then calculated with following contrast vectors: [1 –1 0 0] for pleasant pictures, [1 –1 0 0] for unpleasant pictures, and [0 0 0 1] for VFT.

To reflect the differences more intuitively between groups, the pre-processed data of the region of interest (ROI) were averaged across blocks and subjects. The mean value of oxy-Hb changes during the rest period was subtracted from the block average for baseline correction.

### Statistical analysis

Group comparison of sex was conducted using the chi-square test. Group comparison of other demographic information, clinical and behavioral performance, and condition-wise effects of fNIRS data were compared by *t*-test. Channels with significant differences between groups were defined as ROIs of the corresponding condition. The mean value and SD of averaged beta-estimates within ROI during emotion induction tasks and VFT were calculated. Correlation between HAMD/HAMA scores and the averaged beta-estimates within ROI during emotion induction tasks were computed and between VFT performance and the averaged beta-estimates within ROI during VFT, as well. A correlation coefficient of 0.10 is thought to represent a weak association than that of 0.30 which is a moderate correlation and that of 0.50 is the strongest (Cohen, 2013). Statistical significance was set at *p* < 0.05, uncorrected.

## Results

### Demographic information and physiological assessment

As shown in Table 1, patients with BD and healthy controls did not differ in sex ratio, age, and education level. There were no significant differences in pulse rate and oxygen saturation between groups, indicating a comparable physiological baseline.

**TABLE 1:** Demographic characteristics, clinical information, and VFT performance, beta estimates within region of interest (ROI) during tasks.

	HC (*N* = 45)	BD (*N* = 46)	Group comparison (*P*)
Sex (male/female)	20/25	22/24	0.75
Age (years)	28.69 ± 6.55	30.39 ± 8.35	0.40
Education level (years)	15.07 ± 2.66	15.20 ± 2.34	0.85
Pulse rate (min)	77.83 ± 8.22	79.91 ± 7.64	0.38
Oxygen saturation (%)	0.97 ± 0.01	0.97 ± 0.01	0.41
HAMA total score	0.91 ± 1.01	11.78 ± 5.68	<0.00
HAMD total score	2.69 ± 1.09	18.37 ± 7.26	<0.00
VFT performance	9.97 ± 1.58	6.29 ± 1.72	<0.00
**Beta estimates within ROI (10^∧^-3)**			
Pleasant	0.89 ± 3.70	–6.40 ± 5.60	<0.05
Unpleasant	–12.5 ± 6.00	9.30 ± 7.30	<0.05
VFT	2.63 ± 1.20	–4.50 ± 1.57	<0.05

HAMD, Hamilton depression scale; HAMA, Hamilton anxiety rating scale; VFT performance, correct responses averaged across four blocks of the verbal fluency task; HC, healthy control; and BD, bipolar disorder.

### Assessment of Hamilton depression scale/Hamilton anxiety rating scale and verbal fluency task performance

Patients with BD differ significantly from healthy controls in HAMA total score (*p* < 0.00) and HAMD total score (*p* < 0.00), indicating a higher level of anxiety and depression in patients with BD, as expected. Also, VFT performance was significantly different between BD and HC groups (*p* < 0.00). Patients with BD generated fewer words than healthy controls.

### Comparison of frontal activation between groups

**FIGURE 3:**
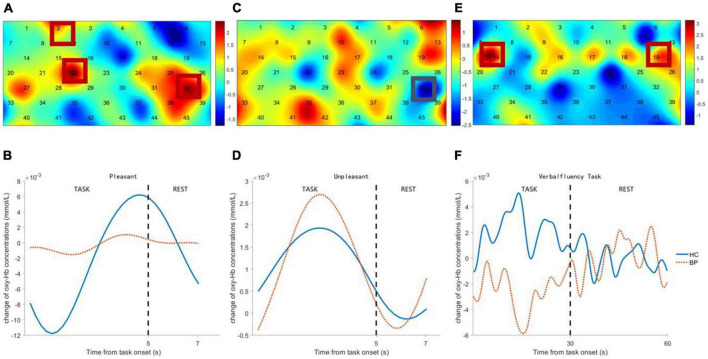
Comparison between bipolar disorder (BD) patients and healthy controls (HCs) of frontal activation and time course of hemodynamic changes evoked by pleasant pictures **(A,B)**, unpleasant pictures **(C,D)**, and verbal fluency task (VFT) task **(E,F)**. The colormap represents the discrepancy (*t*-value) of frontal cortex activation between BD patients and HCs.

Channels in right DLPFC (Ch-2, 22) and left ventrolateral prefrontal cortex (VLPFC) (Ch-32) showed significant differences in viewing pleasant pictures, with lower hemodynamic changes in patients with BD (see Figures 3A,B). Channel 32 (left VLPFC) showed a significant difference during unpleasant picture viewing, with higher hemodynamic changes in patients with BD (see Figures 3C,D). Channels in bilateral DLPFC (Ch-14, 19) showed significant differences during the VFT task, with lower hemodynamic changes in patients with BD (see Figures 3E,F).

### Correlation analysis

Frontal activity evoked by unpleasant pictures was positively correlated with HAMA total scores of the patients with BD and healthy controls (*r* = 0.25, *p* < 0.02) and positively correlated with HAMD total scores, but with marginal significance (*r* = 0.20, *p* = 0.06). There was no significant correlation between frontal activation evoked by pleasant pictures and HAMA total scores (*r* = –0.07, *p* = 0.52), or with HAMD total scores (*r* = –0.15, *p* = 0.16). Frontal activation evoked by VFT was positively correlated with VFT performance (*r* = 0.37, *p* < 0.00) and negatively correlated with HAMA total scores (*r* = –0.26, *p* < 0.02) and HAMD total scores (*r* = –0.27, *p* < 0.02).

## Discussion

The goal of the present study was to probe the abnormal pattern of frontal activity during emotional and cognitive processing in patients with BD. The results presented above support our hypothesis of hypofrontality during verbal fluency with poor cognitive performance in patients with BD and abnormal frontal activity during emotion induction.

### Frontal activity induced by pleasant and unpleasant stimuli in patients with bipolar disorder

In the present study, patients with BD differentiate from the healthy controls in frontal activity induced by emotion-laden stimuli, in a valence-dependent manner. Compared to healthy controls, patients with BD show lower hemodynamic changes in right DLPFC and left VLPFC during viewing pleasant pictures. This attenuated frontal activity is consistent with previous fMRI and fNIRS studies which report decreased inferior frontal gyrus (IFG) activation during emotional tasks in patients with BD (Chen et al., 2011; Hoshi et al., 2011; Frey et al., 2013). In a study that combined lesion and neuroimaging techniques, PFC lesions caused by middle cerebral artery (MCA) stroke induced a deficit of pleasant experience and reduced neural activity in the bilateral dorsal prefrontal cortex and the left superior frontal gyrus (Paradiso et al., 2011).

On the contrary, higher hemodynamic changes in left VLPFC while viewing unpleasant pictures were found in patients with BD as compared to healthy controls, and the hyperactivity was positively correlated with the severity of anxiety/depression. Though the correlation remained at a weak-to-moderate level, partly due to the comparatively large individual difference in frontal activity induced by emotion stimuli, which was also reported in other fNIRS studies concerning neural correlates of emotion induction (Huang et al., 2017). In healthy volunteers, increased hemodynamic activity in PFC induced by negative stimuli was demonstrated in numerous neuroimaging studies, as a result of the salient effect of negative emotion (Glotzbach et al., 2011; Aldhafeeri et al., 2012; Ozawa et al., 2014; Westgarth et al., 2021). In congruent with most neuroimaging studies investigating emotion processing in patients with BD, the present study revealed that negativity bias was more prominent in patients with BD (Altshuler et al., 2008; Frey et al., 2013; Matsubara et al., 2014, 2015; Segar et al., 2021). Moreover, left lateralization of neural activity induced by negative stimuli in patients with BD was also found in our study, in line with previous BD studies showing increased activation in the left frontal regions in the face of negative stimuli (Matsubara et al., 2014). According to the approach-withdrawal hypothesis proposed by Davidson, the left PFC might be involved in the approach component of the motivational system engaged by emotional stimuli (Davidson, 1992).

This valence-dependent PFC hemodynamic response to emotional stimuli in patients with BD was also reported in an fNIRS study with the emotional Stroop paradigm, where patients with BD showed decreased oxy-Hb in the bilateral middle frontal region responding to happy words and increased oxy-Hb in the left inferior frontal region responding to threat words (Matsubara et al., 2014). Taken together, these findings suggest that valence-dependent PFC neural response to emotional stimuli may be a trait marker of altered emotion processing in patients with BD, regardless of the specific emotional task employed. Lima et al. (2018) reviewed studies investigating emotionality in patients with BD and summarized that BD is related to increased negative reactivity and slightly less positive reactivity. Our study partly agreed with Lima et al. that BD relates to increased negative reactivity but decreased positive reactivity. Abnormal emotionality in patients with BD may also arise from maladaptive strategies for regulating emotions, such as negative rumination and dampening of positive emotion, in an automatic manner (Dodd et al., 2019).

### Verbal fluency task performance and neural correlates in patients with bipolar disorder

The present study found attenuated frontal activity in patients with BD, with a reduced number of words generated during VFT. The verbal fluency impairment demonstrated by behavioral performance was in line with previous studies (Raucher-Chene et al., 2017). A storage deficit, as well as impaired retrieval of semantic memory, was found in patients with poor VFT performance (Rossell and David, 2006; Sung et al., 2013). Chang et al. (2011) proposed that functional deficit in patients with BD resulted from impairment of knowledge-based strategies for categorization in semantic memory. Besides language processing, factor analysis shows the executive component involved in VFT (Aita et al., 2019).

In healthy volunteers, prominent activation in frontal regions including the left anterior cingulate gyrus, and the superior, inferior, and medial frontal gyrus, during the VFT task was reported in an fMRI meta-analysis (Wagner et al., 2014). In an fNIRS meta-analysis of studies using VFT to investigate psychiatric disorders, reduced HbO changes in frontal and temporal regions during VFT tasks were found in psychiatric patients including BD compared with healthy controls, as a manifestation of neural inefficiency (Yeung and Lin, 2021). As consistent with previous neuroimaging studies, our present study found hypoactivity in bilateral DLPFC, positively correlated with deterioration of VFT performance and severity of anxiety/depression in patients with BD. The impaired behavioral performance and frontal hypoactivity indicate the functional deficits of frontal regions in processing semantic memory and executive control in patients with BD (Sung et al., 2013; Fu et al., 2018; Aita et al., 2019).

### Limitations

The current study has several limitations. First of all, only patients with BD with depression were recruited, while patients of different mental statuses may behave differently and exhibit distinct neural correlates, especially when mood shifts occur. Second, the drug effect was not ruled out in our findings, while patients were taking multiple antidepressants or stabilizers. However, there was no direct evidence of medication effects on oxy-Hb concentrations in patients with BD. According to a review of medication effects in neuroimaging studies of BD, psychotropic medication had a limited impact on fMRI findings (Hafeman et al., 2012). Therefore, we infer that medication may not likely contribute to the different patterns of hemodynamic activity observed between patients with BD and healthy controls in the present study. Third, emotional and cognitive processing was investigated separately in our study. Further investigations are needed to develop an integrated design to explore the intricate link between emotionality and cognition in BD.

## Conclusion

The present study found distinct hemisphere activity in the prefrontal region during emotional and cognitive processing between patients with BD and healthy controls. Patients with BD exhibit valence-dependent PFC hemodynamic response to emotional stimuli, with bilateral frontal hypoactivity indicating decreased positive reactivity and left frontal hyperactivity indicating increased negative reactivity. Together with the impaired performance and hypofrontality during VFT, dysfunction of cognition and emotionality in patients with BD probed by fNIRS would be a potential biomarker in clinical assessment.

## Data availability statement

The raw data supporting the conclusions of this article will be made available by the authors, without undue reservation.

## Ethics statement

The studies involving human participants were reviewed and approved by Ethics Committee of Yuquan Hospital. The patients/participants provided their written informed consent to participate in this study.

## Author contributions

MM: data pre-processing and analysis, manuscript writing, and revision. GC: study design and data collection. KF: patient diagnosis and data collection. DX, XH, and CS: manuscript writing guidance and revision. PL: patient diagnosis and manuscript writing guidance. All authors contributed to the article and approved the submitted version.

## Conflict of interest

The authors declare that the research was conducted in the absence of any commercial or financial relationships that could be construed as a potential conflict of interest.

## Publisher’s note

All claims expressed in this article are solely those of the authors and do not necessarily represent those of their affiliated organizations, or those of the publisher, the editors and the reviewers. Any product that may be evaluated in this article, or claim that may be made by its manufacturer, is not guaranteed or endorsed by the publisher.
